# Retrospective Validation of the Brain Injury Guidelines at a Single Community Institution

**DOI:** 10.7759/cureus.108352

**Published:** 2026-05-06

**Authors:** Gianmarino C Gianfrate, Kathryn Ogborn, Stacy A Lane, Michael Sippel, Gregory S Huang

**Affiliations:** 1 General Surgery, Mercy Health - St. Elizabeth Youngstown Hospital, Youngstown, USA; 2 General surgery, Mercy Health - St. Elizabeth Youngstown Hospital, Youngstown, USA; 3 Trauma Surgery, Mercy Health - St. Elizabeth Youngstown Hospital, Youngstown, USA; 4 Trauma, Critical Care, and General Surgery Services, Mercy Health - St. Elizabeth Youngstown Hospital, Youngstown, USA

**Keywords:** brain injury guidelines (big), community trauma centers, neurosurgery technique, practice management guidelines, systemic anticoagulation, traumatic brain injury

## Abstract

Background

Traumatic brain injury (TBI) continues to increase in incidence and resource utilization. The Brain Injury Guidelines (BIG) aim to standardize management of isolated blunt TBI and reduce unnecessary interventions; however, adoption in community and regional trauma centers remains limited. We sought to validate BIG criteria at a regional Level I trauma center.

Methods

We performed a retrospective review of adults with isolated blunt TBI and positive head CT findings from 2018 to 2019. Patients requiring emergent surgery or with polytrauma were excluded. Patients were stratified into BIG 1-3 categories. Outcomes included neurological deterioration (GCS change >2), neurosurgical intervention (Subdural or intraparenchymal Bolt and Craniotomy or craniectomy), mortality, hemorrhage progression, and length of stay. Multivariable logistic regression identified predictors of adverse outcomes.

Results

Among 557 patients (BIG 1: 30.5%, BIG 2: 19.7%, BIG 3: 49.7%), median age was 77 years, and falls accounted for 86% of injuries. Overall rates of neurological deterioration, neurosurgical intervention, and mortality were 5.4% (n=557), 1.6% (n=557), and 3.6% (n=557), respectively. No BIG 1 patients required neurosurgical intervention, and adverse outcomes were rare. Hemorrhage progression occurred in 12.7% (n=534), most frequently in BIG 3 (17.8%, n=534). Independent predictors of adverse outcomes included BIG 3 classification (OR 6.8), age ≥75 years (OR 2.9), anticoagulation use (OR 2.2), and hemorrhage progression (OR 5.1). Length of stay increased significantly with higher BIG classification (p<0.001).

Conclusions

The BIG criteria are safe and effective for risk stratification in a regional Level I trauma center. BIG 1 patients demonstrated minimal risk and may not require routine admission, repeat imaging, or neurosurgical consultation. Broader implementation may reduce resource utilization and improve care efficiency without compromising outcomes.

## Introduction

Traumatic brain injury (TBI) remains a major contributor to morbidity, mortality, and healthcare utilization, with rising incidence over the past two decades driven largely by an aging population and increased use of anticoagulant and antiplatelet therapies [1]. Despite the high prevalence of isolated blunt TBI, management strategies remain variable, often resulting in routine hospital admission, repeat imaging, and neurosurgical consultation regardless of injury severity.

The Brain Injury Guidelines (BIG), first introduced by Joseph et al. in 2013, were developed to provide a standardized, risk-stratified approach to the management of TBI based on clinical and radiographic findings [2]. These guidelines have been validated across multiple retrospective and prospective cohorts, demonstrating the ability to safely reduce unnecessary resource utilization, particularly among low-risk patients. Subsequent adaptations, including modified BIG (mBIG) protocols, have further supported their clinical applicability.

Despite growing evidence, adoption of BIG-based protocols has been inconsistent, particularly in regional and community trauma centers [3-6]. Concerns regarding heterogeneity in TBI presentation, variability in definitions, and limited generalizability of existing studies have contributed to hesitancy in implementation [7,8]. As a result, many institutions continue to follow more conservative management strategies, contributing to increased healthcare costs and system burden [9].

The primary objective of this study was to externally validate the Brain Injury Guidelines (BIG) criteria by assessing their ability to stratify patients with traumatic intracranial hemorrhage according to risk of adverse clinical outcomes. Secondary objectives were to evaluate healthcare resource utilization across BIG categories, including rates of ICU admission, repeat neuroimaging, neurosurgical consultation, and hospital length of stay, and identify clinical and radiographic predictors of adverse outcomes, including neurological deterioration, need for neurosurgical intervention, and in-hospital mortality. By clearly distinguishing validation of the BIG criteria from analyses of resource utilization and predictors, the study aims to assess both clinical performance and operational implications of BIG implementation.

## Materials and methods

This was a retrospective observational cohort study conducted at a single Level I trauma center in northeast Ohio from January 1st, 2018, to January 1st, 2019. Institutional Review Board approval was obtained prior to study initiation, with a waiver of informed consent due to the retrospective nature of the study. All TBI patients were of age ≥ 18 years, presented following blunt traumatic injury, had evidence of traumatic intracranial hemorrhage on initial head CT scan, and had the availability of complete clinical, imaging, and outcome data. Penetrating head injury, transfer from an outside hospital with incomplete records, death on arrival or prior to initial CT imaging, and pre-existing neurosurgical intervention during the index hospitalization were excluded.

After institutional review board approval, eligible patients were identified by the Trauma Registry. Variables collected via medical records and the Trauma Registry included: demographics (age and sex), patients' antiplatelet and anticoagulation use, requirement of anticoagulation/antiplatelet reversal, anti-seizure medication, vitals on presentation, Glasgow Coma Scale (GCS) at presentation, GCS at 15 minutes after presentation and GCS at discharge, intoxication with alcohol, urine drug screen results, mechanism of injury, initial neurosurgical evaluation, follow up neurosurgical evaluation after hospital stay, initial CT head scan, repeat CT head scan, outpatient CT scan of head if performed, Injury Severity Score (ISS), hospital disposition, hospital length of stay, and ICU length of stay.

Patients were retrospectively categorized into BIG categories (BIG 1-3) based on presenting clinical and radiographic findings [2]. Patients with BIG 1 (minor head injury) had a normal neurologic examination finding, were not on any antiplatelet or anticoagulation medications, and had minuscule findings on initial head CT scan (ICH < 4mm and no skull fracture). BIG 2 category was composed of moderately injured patients with non-displaced skull fractures. Patients categorized as BIG 3 were on antiplatelet or anticoagulation medications, had an abnormal neurologic examination finding, and concerning CT scan findings (displaced skull fracture, ICH > 8mm, midline shift). Non-examinable patients, intubated patients, and patients with more than one CT finding were also categorized as BIG 3.

After each patient was categorized into one of the three groups, each group’s outcomes were measured and compared. These outcomes were then compared with the already published BIG guidelines [2]. Data was entered into Excel (Microsoft Corp., Redmond, WA, USA) and transferred into the SPSS 23.0 (IBM Corp., Armonk, NY, USA) statistical program. Analysis included descriptive statistics, ANOVA, means, and standard deviations.

Statistical analyses were performed to address both the primary objective of validating the Brain Injury Guidelines (BIG) criteria and secondary objectives related to resource utilization and predictors of adverse outcomes. Data distribution for continuous variables was assessed using visual inspection and normality testing. Because most continuous variables were non-normally distributed, results are reported as medians with interquartile ranges and compared using Mann-Whitney U tests; categorical variables were compared using chi-square or Fisher's exact tests, as appropriate. Multivariable logistic regression was used to identify independent predictors of adverse outcomes, with candidate variables selected based on clinical relevance and univariable associations. Model assumptions, including multicollinearity and goodness of fit, were assessed prior to final model selection. Adjusted odds ratios with 95% confidence intervals are reported. All tests were two-sided, and a p-value < 0.05 was considered statistically significant. Analyses were performed using appropriate statistical software, with adjustment for multiple comparisons where indicated. This methodological approach allows for rigorous validation of the BIG criteria while also evaluating associated healthcare resource utilization and clinical risk factors.

## Results

Study population and demographics

Between April 2018 and December 2019, 557 patients with traumatic intracranial hemorrhage met the inclusion criteria. Patients were stratified into Brain Injury Guideline (BIG) categories: BIG 1 (n = 170, 30.5%), BIG 2 (n = 110, 19.7%), and BIG 3 (n = 277, 49.7%).

The median age was 77 years (IQR 64-86), and 311 patients (55.8%) were female. Sex distribution did not differ across BIG categories (χ²(2, N = 557) = 1.66, p = .44, Cramér’s V = .05). Racial distribution was similarly comparable between groups (χ²(4, N = 557) = 0.36, p = .99, Cramér’s V = .02).

Falls were the predominant mechanism of injury across all categories (BIG 1: 89.4%, BIG 2: 86.4%, BIG 3: 83.8%), followed by motor vehicle trauma and assault, with no significant difference by BIG classification (χ²(6, N = 557) = 4.00, p = .68, Cramér’s V = .06).

The median Injury Severity Score (ISS) was 9 (IQR 5-17), and the median Glasgow Coma Scale (GCS) score at presentation was 15 (IQR 14-15).

Anticoagulant or antiplatelet therapy was present in 327 patients (58.7%), with no difference across BIG categories (χ²(2, N = 557) = 2.16, p = .34, Cramér’s V = .06). Among anticoagulated patients, 102 (31.2%) received reversal therapy (Table 1).

**Table 1:** Baseline Characteristics by BIG Category IQR: Interquartile Range; ISS: Injury Severity Score; GCS: Glasgow Coma Scale

Characteristic	BIG 1 (n=170)	BIG 2 (n=110)	BIG 3 (n=277)	P Value
Age, median (IQR), y	71 (56-81)	78 (67-85)	81 (72-88)	0.001
Female sex, No. (%)	88 (51.8)	64 (58.2)	159 (57.4)	0.42
Race, No. (%)				0.78
White	160 (94.1)	103 (93.6)	261 (94.2)	
Black	8 (4.7)	5 (4.5)	13 (4.7)	
Other	2 (1.2)	2 (1.8)	3 (1.1)	
Mechanism, No. (%)				0.08
Fall	152 (89.4)	95 (86.4)	232 (83.8)	
Motor vehicle trauma	11 (6.5)	9 (8.2)	24 (8.7)	
Assault	3 (1.8)	3 (2.7)	13 (4.7)	
Other	4 (2.4)	3 (2.7)	8 (2.9)	
ISS, median (IQR)	5 (4-9)	9 (5-14)	16 (10-21)	0.001
Admission GCS, median (IQR)	15 (15-15)	15 (14-15)	14 (13-15)	0.001
Anticoagulation, No. (%)	92 (54.1)	66 (60.0)	169 (61.0)	0.32
Reversal administered, No. (%)	25 (27, n=92)	21 (31.8, n=66)	56 (33.1, n=169)	0.31

Hemorrhage characteristics

Subdural hemorrhage (SDH) was the most common hemorrhage type (58.5%), followed by subarachnoid hemorrhage (33.4%), intraparenchymal hemorrhage (5.9%), and epidural hemorrhage (2.2%). Median hemorrhage size was 4 mm (IQR 2-7 mm), and loss of consciousness occurred in 56.0% of patients.

Hemorrhage type differed significantly across BIG categories (χ²(6, N = 557) = 14.10, p = .029, Cramér’s V = .11). Midline shift was also significantly associated with BIG classification (χ²(2, N = 557) = 44.94, p < .001, Cramér’s V = .28).

There were no significant differences in loss of consciousness (χ²(2, N = 557) = 4.36, p = .11, Cramér’s V = .09) or skull fracture (χ²(2, N = 557) = 4.80, p = .091, Cramér’s V = .09) across groups (Table 2).

**Table 2:** Hemorrhage Characteristics and Imaging Findings IQR: Interquartile Range

Characteristic	Overall (n=557)	BIG 1 (n=170)	BIG 2 (n=110)	BIG 3 (n=277)	P Value
Hemorrhage type, No. (%)					0.001
Subdural Hemorrhage (SDH)	326 (58.5)	88 (51.8)	68 (61.8)	170 (61.4)	
Subarachnoid Hemorrhage (SAH)	186 (33.4)	74 (43.5)	34 (30.9)	78 (28.2)	
Intraparenchymal Hemorrhage (IPH)	33 (5.9)	6 (3.5)	6 (5.5)	21 (7.6)	
Epidural Hemorrhage (EDH)	12 (2.2)	2 (1.2)	2 (1.8)	8 (2.9)	
Hemorrhage size, median (IQR), mm	4 (2-7)	2 (1-3)	5 (3-6)	8 (5-13)	0.001
Loss of consciousness, No. (%)	312 (56.0)	84 (49.4)	64 (58.2)	164 (59.2)	0.11
Midline shift, No. (%)	48 (8.6)	0 (0)	2 (1.8)	46 (16.6)	0.001
Skull fracture, No. (%)	42 (7.5)	7 (4.1)	8 (7.3)	27 (9.7)	0.08

Repeat imaging and hemorrhage progression

Repeat head CT was performed in 534 patients (95.9%) at a median of 18 hours (IQR 12-24). Hemorrhage progression occurred in 68 patients (12.7%), defined as increased hemorrhage size, new hemorrhage, or worsening midline shift. Among those with progression, the median increase in hemorrhage size was 3 mm (IQR 2-5 mm). Complete resolution was observed in 132 patients (24.7%).

Hemorrhage progression differed significantly by BIG category (χ²(2, N = 534) = 16.70, p < .001, Cramér’s V = .18), as did increased hemorrhage size (χ²(2, N = 534) = 11.59, p = .003, Cramér’s V = .15). Complete resolution was also significantly associated with BIG classification (χ²(2, N = 534) = 49.04, p < .001, Cramér’s V = .30).

No differences were observed in new hemorrhage (χ²(2, N = 534) = 4.06, p = .13, Cramér’s V = .09) or increased midline shift (χ²(2, N = 534) = 5.29, p = .071, Cramér’s V = .10) (Table 3).

**Table 3:** Repeat Imaging Findings and Hemorrhage Progression IQR: Interquartile Range

Finding	Overall (n=534)	BIG 1 (n=164)	BIG 2 (n=106)	BIG 3 (n=264)	P Value
Repeat CT performed, No. (%)	534 (95.9, n=557)	164	106	264	0.72
Time to repeat CT, median (IQR), h	18 (12-24)	20 (15-24)	18 (12-24)	16 (10-22)	0.01
Hemorrhage progression, No. (%)	68 (12.7)	7 (4.3)	14 (13.2)	47 (17.8)	0.001
Type of progression, No. (%)					
Increased size	44 (8.2)	4 (2.4)	9 (8.5)	31 (11.7)	0.002
New hemorrhage	19 (3.6)	2 (1.2)	4 (3.8)	13 (4.9)	0.11
Increased shift	14 (2.6)	1 (0.6)	2 (1.9)	11 (4.2)	0.04
Complete resolution, No. (%)	132 (24.7)	71 (43.3)	26 (24.5)	35 (13.3)	0.001

Clinical outcomes

The median hospital length of stay was 2 days (IQR 1-4), and the median ICU length of stay was 0 days (IQR 0-2). Neurological deterioration occurred in 30 patients (5.4%), and neurosurgical intervention was required in nine patients (1.6%).

Neurological deterioration varied significantly across BIG categories (χ²(2, N = 557) = 10.69, p = .005, Cramér’s V = .14), as did neurosurgical intervention (χ²(2, N = 557) = 9.25, p = .010, Cramér’s V = .13) and medical complications (χ²(2, N = 557) = 7.13, p = .028, Cramér’s V = .11).

In-hospital mortality occurred in 20 patients (3.6%) and differed significantly by BIG classification (χ²(2, N = 557) = 7.68, p = .021, Cramér’s V = .12).

At discharge, 86.2% of patients had favorable outcomes (home or acute rehabilitation), while 10.2% were discharged to hospice or long-term care. Discharge disposition also varied significantly across groups (χ²(8, N = 557) = 29.66, p < .001, Cramér’s V = .16) (Table 4).

**Table 4:** Clinical Outcomes by BIG Category LOS: Length of Stay; ICU: Intensive Care Unit; LTAC: Long-Term Acute Care

Outcome	Overall (n=557)	BIG 1 (n=170)	BIG 2 (n=110)	BIG 3 (n=277)	P Value
Hospital LOS, median (IQR), d	2 (1-4)	1 (1-2)	2 (1-3)	3 (2-6)	0.001
ICU LOS, median (IQR), d	0 (0-2)	0 (0-0)	0 (0-1)	2 (0-5)	0.001
Neurological deterioration, No. (%)	30 (5.4)	2 (1.2)	5 (4.5)	23 (8.3)	0.006
Neurosurgical intervention, No. (%)	9 (1.6)	0 (0)	0 (0)	9 (3.2)	0.01
Medical complications, No. (%)	46 (8.3)	7 (4.1)	8 (7.3)	31 (11.2)	0.02
In-hospital mortality, No. (%)	20 (3.6)	2 (1.2)	2 (1.8)	16 (5.8)	0.03
Discharge disposition, No. (%)					0.001
Home	414 (74.3)	146 (85.9)	84 (76.4)	184 (66.4)	
Acute rehabilitation	66 (11.8)	17 (10.0)	14 (12.7)	35 (12.6)	
Skilled nursing/LTAC	34 (6.1)	3 (1.8)	7 (6.4)	24 (8.7)	
Hospice	23 (4.1)	2 (1.2)	3 (2.7)	18 (6.5)	
Deceased	20 (3.6)	2 (1.2)	2 (1.8)	16 (5.8)	

Follow-up outcomes

Neurosurgical follow-up at 20-30 days was completed in 430 patients (77.2%). Among these, 87.0% demonstrated complete resolution or improvement on imaging, 10.2% had stable findings, and 2.8% had persistent or worsening hemorrhage.

Follow-up imaging outcomes differed significantly across BIG categories (χ²(6, N = 430) = 46.42, p < .001, Cramér’s V = .23). However, 7-day return visits (χ²(2, N = 430) = 1.98, p = .37, Cramér’s V = .07) and readmissions (χ²(2, N = 430) = 4.40, p = .11, Cramér’s V = .10) did not differ between groups (Table 5).

**Table 5:** Follow-up Outcomes

Outcome	Overall (n=430)	BIG 1 (n=155)	BIG 2 (n=87)	BIG 3 (n=188)	P Value
Follow-up completed, No. (%)	430 (77.2, n=557)	155	87	188	0.001
Follow-up imaging findings, No. (%)					0.001
Complete resolution	278 (64.7)	124 (80.0)	58 (66.7)	96 (51.1)	
Improved	96 (22.3)	26 (16.8)	23 (26.4)	47 (25.0)	
Stable	44 (10.2)	5 (3.2)	5 (5.7)	34 (18.1)	
Worse	12 (2.8)	0 (0)	1 (1.1)	11 (5.9)	
7-day return visit, No. (%)	25 (4.5)	6 (3.5)	5 (4.5)	14 (5.1)	0.68
Readmission required, No. (%)	9 (1.6)	1 (0.6)	1 (0.9)	7 (2.5)	0.23

Predictors of adverse outcomes

Multivariable logistic regression identified independent predictors of adverse outcomes (neurological deterioration, neurosurgical intervention, or death). Age ≥75 years (OR 2.9, 95% CI 1.5-5.8, p = .002), BIG 3 classification (OR 6.8, 95% CI 3.1-14.9, p < .001), anticoagulation use (OR 2.2, 95% CI 1.2-4.3, p = .02), and hemorrhage progression (OR 5.1, 95% CI 2.5-10.4, p < .001) were independently associated with adverse outcomes.

Initial GCS score and hemorrhage type were not independent predictors after adjustment (Table 6).

**Table 6:** Multivariable Predictors of Adverse Outcomes BIG: Brain Injury Guidelines; ISS: Injury Severity Score

Variable	Odds Ratio (95% CI)	P Value
Age ≥75 years	2.9 (1.5-5.8)	0.002
BIG category		
BIG 1	Reference	-
BIG 2	3.4 (1.3-8.9)	0.01
BIG 3	6.8 (3.1-14.9)	0.001
Anticoagulation use	2.2 (1.2-4.3)	0.02
Hemorrhage progression	5.1 (2.5-10.4)	0.001
ISS ≥16	1.9 (0.9-3.8)	0.08
Loss of consciousness	1.5 (0.8-2.9)	0.28

## Discussion

Our findings closely mirror the original Brain Injury Guidelines (BIG) framework and its subsequent validation studies, particularly with respect to treatment stratification and clinical outcomes [2-4]. Consistent with prior reports, patients classified as BIG 1 in our cohort experienced no neurosurgical interventions, extremely low rates of neurological deterioration, and no clinically meaningful benefit from repeat imaging or inpatient admission. These results directly support the original BIG treatment recommendations, which advocate for observation without routine hospitalization, repeat CT imaging, or mandatory neurosurgical consultation in this low-risk population [2-5]. Given that these patients were not on antiplatelet or anticoagulant therapy, the risk of hemorrhage progression was predictably low, further reinforcing the safety of a conservative, outpatient-focused approach.

In contrast, patients categorized as BIG 2 and BIG 3 demonstrated progressively greater injury severity, higher Injury Severity Scores, and increased rates of hemorrhage progression and adverse outcomes. These findings support continued adherence to guideline-based escalation of care in these groups, including hospital admission, repeat imaging, and neurosurgical consultation. This stepwise approach to management highlights the strength of the BIG framework in aligning treatment intensity with patient-specific risk [2-4].

To improve clarity of our study objectives, we explicitly distinguish two complementary aims: (1) to evaluate the safety and real-world applicability of BIG-based risk stratification in a regional trauma population, and (2) to assess associated patterns of resource utilization, including admission and repeat CT imaging. Framed this way, our results demonstrate that while higher-risk groups appropriately consume greater resources, low-risk patients (BIG 1) derive no measurable clinical benefit from routine escalation of care, supporting a more selective approach.

When compared with broader national and society-based practice management guidelines (PMGs), our data underscores an important distinction. Many existing guidelines remain intentionally conservative, often recommending routine repeat imaging and specialist consultation for most patients with intracranial hemorrhage [2,4]. In contrast, the BIG framework offers a more granular, risk-stratified approach. Our results support this more selective strategy, demonstrating that adherence to BIG criteria can safely reduce unnecessary interventions in low-risk patients without compromising outcomes. These findings are consistent with prior studies by Joseph et al. and others, as well as with emerging modified BIG (mBIG) protocols, which have gained increasing acceptance and validation within the trauma community, including endorsement by the American Association for the Surgery of Trauma [2-4]. Notably, recent studies have also explored the application of BIG-based frameworks in pediatric trauma populations, demonstrating similar potential for safe risk stratification and reduction in unnecessary imaging and resource utilization [10-12].

Importantly, our study reinforces that implementation of BIG-based treatment recommendations can extend beyond large academic centers and be safely applied within regional Level I trauma centers [5,6]. While system-level benefits such as reduced resource utilization and decreased healthcare burden are notable, the primary implication of our findings remains clinical: appropriate identification of low-risk patients who can safely avoid unnecessary interventions, while ensuring that higher-risk patients receive timely and appropriate escalation of care [7,13-15]. Our institutional adaptation, which closely aligns with modified BIG principles, emphasizes accurate capture of BIG 1 patients while maintaining vigilance in higher-risk groups. The expanding evaluation of BIG criteria across diverse populations, including pediatric cohorts, further supports the adaptability and broader applicability of this risk-stratified approach [7,10,15].

Taken together, these results support broader adoption of BIG-informed practice management strategies, helping to bridge the gap between established evidence and real-world clinical application across diverse healthcare settings.

This study has several limitations. First, its retrospective, single-center design introduces the potential for selection bias and limits generalizability, particularly given the predominantly elderly, fall-related mechanism of injury in our cohort. Additionally, the cohort had a median age of 77 years, making it older than those in many larger trials and potentially limiting comparisons to younger trauma populations. Second, although outcomes were clearly defined, follow-up was incomplete, with approximately 23% of patients lacking outpatient data, which may underestimate delayed complications. Third, institutional practice patterns, including the high rate of repeat CT imaging, may not reflect strict adherence to BIG-based management and limit conclusions regarding resource utilization. Additionally, variability in radiographic interpretation and clinical documentation may have influenced patient classification. Finally, while multivariable analysis was performed, residual confounding cannot be excluded. Despite these limitations, our findings are consistent with prior validation studies and support the safe application of BIG criteria in a regional trauma setting.

## Conclusions

This study demonstrates that the Brain Injury Guidelines can be safely and effectively applied within a regional Level I trauma center, with findings consistent with prior validations at large academic institutions. Patients classified as BIG 1 experienced minimal risk of clinical deterioration, required no neurosurgical intervention, and derived little benefit from routine hospitalization or repeat imaging. Adoption of a guideline-driven approach has the potential to reduce unnecessary resource utilization, limit healthcare costs, and alleviate system strain without compromising patient safety. Importantly, these results support broader implementation of BIG-based protocols in community and regional settings, helping to bridge the gap between academic research and real-world practice. Continued efforts toward standardization, multicenter collaboration, and data sharing will be essential to further refine TBI management and optimize outcomes across diverse healthcare environments.
